# Wenyang Huazhuo Tongluo formula inhibits fibrosis via suppressing Wnt/β-catenin signaling pathway in a Bleomycin-induced systemic sclerosis mouse model

**DOI:** 10.1186/s13020-018-0175-z

**Published:** 2018-03-27

**Authors:** Qian Wang, Wenhua Zang, Li Han, Lei Yang, Songshan Ye, Jingfeng Ouyang, Chaoyun Zhang, Yuefeng Bi, Cuiyue Zhang, Hua Bian

**Affiliations:** 1 Zhang Zhongjing College of Chinese Medicine, Nanyang Institute of Technology, Changjiang Road 80, Nanyang, 473004 Henan China; 2 Henan Key Laboratory of Zhang Zhongjing Formulae and Herbs for Immunoregulation, Nanyang Institute of Technology, Nanyang, Henan China; 3 Experimental Research Center, China Academy of Chinese Medical Sciences, Beijing, China; 4 School of Pharmaceutical Sciences, Zhengzhou University, Zhengzhou, Henan China

**Keywords:** Wenyang Huazhuo Tongluo formula, SSc mouse model, Anti-fibrosis, Wnt/β-catenin signaling pathway

## Abstract

**Background:**

Systemic sclerosis (SSc) is an autoimmune disease characterized by fibrosis of the skin and internal organs. So far, no Western medicine treatment can completely inhibit or reverse the progress of SSc, while at the same time, our previous series of studies have shown that the treatment of SSc by the Wenyang Huazhuo Tongluo formula (WYHZTL), a Chinese herbal decoction, shows a delightful prospect. The aim of this study is to further investigate the mechanism of anti-fibrosis of WYHZTL formula in SSc mouse model.

**Methods:**

The Bleomycin-induced SSc mouse model was treated with saline (BLM), high-dosage of WYHZTL formula (WYHZTL-H), medium-dosage of WYHZTL formula (WYHZTL-M), low-dosage of WYHZTL formula (WYHZTL-L) and XAV-939, a small molecule inhibitor of Wnt/β-catenin signaling pathway, by the intragastric administration and intraperitoneal injection, respectively. The mRNA and protein levels of Wnt/β-catenin signaling pathway associated genes, fibrosis markers and histopathology were detected by reverse transcription-quantitative polymerase chain reaction, Western blotting and hematoxylin/eosin-staining. The levels of Wnt1, CTGF and DKK1 protein in serum were detected by enzyme-linked immunosorbent assay.

**Results:**

Compared with BLM group, the WYHZTL formula and XAV-939 could significantly inhibit the thickness of the skin tissue of the SSc mouse model. The mRNA expression levels of GSK3β and DKK1 in the WYHZTL formula and XAV-939-treated group were significantly higher than those in the BLM group, while Wnt1, β-catenin, TCF4, cyclin D1, survivin, VEGF, CTGF, FN1, collagen I/III were decreased. Compared with BLM group, the protein expression levels of GSK3β and DKK1 in the WYHZTL formula and XAV-939-treated group were upregulated, while Wnt1, β-catenin, cyclin D1, survivin, CTGF, FN1, collagen I/III were downregulated. WYHZTL formula and XAV-939 could inhibit expression of Wnt1 and CTGF, but promoted DKK1 in serum. Furthermore, WYHZTL-H seemed more effective than WYHZTL-M and/or XAV-939 on regulating Wnt1, β-catenin, TCF4, GSK3β, DKK1, cyclin D1, survivin, VEGF, CTGF, FN1 and collagen I/III.

**Conclusion:**

This present study demonstrates that WYHZTL formula has anti-fibrosis effect in Bleomycin-induced SSc mouse model in a dosage-dependent manner, and the molecular mechanism may be related to the inhibition of Wnt/β-catenin signaling pathway.

**Electronic supplementary material:**

The online version of this article (10.1186/s13020-018-0175-z) contains supplementary material, which is available to authorized users.

## Background

Systemic sclerosis (SSc) is an autoimmune disease characterized by fibrosis of the skin and internal organs. Fibrosis is a typical pathological feature of SSc, which is closely related to its prognosis and mortality [1, 2]. So far, there is no drug that can completely inhibit or reverse the progress of SSc, neither the plasma exchange treatment indicating by clinical score [3]. Encouragingly, hematopoietic stem cell transplantation shows a certain effect on the treatment of SSc, but needs a long way for clinical applications [4].

Studies have shown that the occurrence, development and prognosis of SSc fibrosis depends on the “net effect” of the synthesis and degradation processes of extracellular matrix (ECM). Fibrosis occurs when collagen fibers are excessively synthesized and relatively less degraded [5, 6]. Wnt signal pathway plays an important role in regulating the adhesion, migration, epithelial transformation and growth of cells, and maintaining the stability of tissues and organs [7, 8]. When the Wnt/β-catenin signaling pathway, the classical Wnt signaling pathway, is inactive, Axin, APC, GSK3β and CK1 in the cytoplasm form a degraded complex that binds to β-catenin, leading β-catenin degraded by proteasomes. On the other hand, when the Wnt protein binds to the receptors of Frizzled family on the cell membrane, the Wnt/β-catenin signaling pathway is activated, causing the accumulation of β-catenin in the cytoplasm to promote the translocation of β-catenin to the nucleus to form transcriptional activation complex with TCF/LEF family proteins, followed by the upregulation of a series of target genes, such as c-myc and cyclin D1 and so on [9, 10].

Previous studies showed that Wnt/β-catenin signaling pathway was closely related to fibrosis in skin, lung, liver and kidney [11–15], which played an important role in the pathogenesis of SSc fibrosis. In the process of SSc fibrosis, Wnt/β-catenin signaling pathway was overactive, and the expression level of target genes, such as cyclinD1, VEGF, CTGF and ET-1 are abnormal [16, 17]. Compared with normal subjects, the accumulation of β-catenin in the SSc skin fibroblasts was increased, which resulted in higher transcription level of the target genes. At the same time, the expression levels of Wnt-1 and Wnt-10b were increased in the fibrotic skin and lung tissue [18]. In the Bleomycin-induced fibrosis model or Tsk mice, Wnt/β-catenin signaling pathway was also overactivated [18–20]. Similarly, Liu et al. [21] found that the expression level of cyclin D1 and TGF-β in pulmonary fibrosis were elevated in the Bleomycin-induced animal model. On the contrary, Dkk1 inhibited Wnt/β-catenin signaling pathway by binding to LRP5 and LRP6, the Wnt receptor complex [22]. Akhmetshina et al. [23, 24] found that DKK1 expression was absent in SSc dermal fibroblasts and also significantly reduced in pulmonary fibrosis. The animal experiments showed that DKK1 overexpression inhibited Bleomycin-induced fibrosis [18]. By injecting DKK siRNA into mouse ears, scientists found that expression level of DKK was decreased, and dermal was thickening, which accompanied by increased fibroblast growth and β-catenin expression [25]. All the results showed that DKK1 deletion was one of the causes of Wnt/β-catenin signaling pathway overactivation and played an important role in the pathogenesis of SSc.

The current western medicine treatment of SSc is not satisfactory, so it is essential to explore another treatment option. In the traditional Chinese medicine treatment of SSc, Wenyangchubi formula and Yiqihuoxue formula were representative examples. Studies have shown that Wenyangchubi formula could reduce the expression of connective tissue growth factor (CTGF) and collagen I, improve the skin fibrosis of SSc mouse model [26]. And Yiqihuoxue formula could inhibit the expression of collagen in SSc dermal fibroblasts and TGF-β1-induced NIH/3T3 fibroblasts by regulating TGF-β/smad3 signaling pathway [27]. In 2000, our team invented Wenyang Huazhuo Tongluo formula (WYHZTL formula, Patent No. CN201310351880.2), and applied to the treatment of SSc. Our previous studies have shown that WYHZTL formula could effectively improve the skin score of SSc patients, Renault phenomenon and nail fold microcirculation, reduce plasma endothelin, FVC, DLco and other indicators [28]. In vitro, WYHZTL formula could inhibit the expression of cyclin D1, survivin, TGF-β1 and collagen I/III in skin fibroblasts of SSc patients [29, 30]. WYHZTL formula promoted MMP-9 secretion, inhibited TIMP-1 production, regulated MMP-9/TIMP-1 imbalance, and reduced the serum PIIINP levels while upregulated ICTP levels in SSc patients, thereby reducing fibrosis [31]. However, the mechanism of WYHZTL formula in the treatment of SSc fibrosis based on Wnt/β-catenin signaling pathway has not been reported.

The aim of this study was to further explore the molecular mechanism of anti-SSc fibrosis of WYHZTL formula, and investigate the effect of WYHZTL formula on Wnt/β-catenin signaling pathway in SSc mouse model.

## Methods

The Minimum Standards of Reporting Checklist contains details of the experimental design, and resources used in this study (Additional file 1).

### Composition and preparation of Wenyang Huazhuo Tongluo formula

WYHZTL formula contains Herba Epimedii, Radix Astragali membranacei, Herba Glechomae longitubae, Ramulus Cinnamomi cassiae, Radix Dioscoreae oppositae, Semen Sinapis lbae, Radix Codonopsitis pilosulae, Fasciculus vascularis Luffae, Radix Rehmanniae praeparata and Capparis zeylanica Linn. The preparation procedure of WYHZTL was performed as our previous protocol [29]. The ingredients of WYHZTL formula were purchased from the First Affiliated Hospital of Nanyang Institute of Technology.

### The establishment of Bleomycin-induced systemic sclerosis mouse model

Specific pathogen-free (SPF) C57BL/6 mice were purchased from Beijing Vital River Laboratory Animal Technology Co., Ltd. (Beijing, China). By local injection of Bleomycin in 7-week-old female mice for 3 weeks, the skin fibrosis was induced. Briefly, Bleomycin dissolved in sterile saline to a concentration of 200 μg/ml, and subcutaneous injection of 100 μl of Bleomycin to the restricted area of the upper back each day [27]. 100 μl of phosphate buffer saline (PBS) was subcutaneously injected as controls. All the animal protocols were approved by Zhang Zhongjing College of Chinese Medicine, Nanyang Institute of Technology, China.

### The experimental group and drug treatment regimens

Eight mice on a C57BL/6 background were randomly selected as normal groups. Then, the Bleomycin-induced SSc mouse models were randomly divided into five groups, and each group has eight mice. The five groups were named as Bleomycin group (BLM), WYHZTL formula low-dosage group (BLM + WHHZTL-L), WYHZTL formula medium-dosage group (BLM + WYHZTL-M), WYHZTL formula high-dosage group (BLM + WYHZTL-H), and XAV-939 group (BLM + XAV-939), respectively. The PBS group (PBS) received PBS administered daily by subcutaneous injection into defined areas of upper back of normal C57BL/6 mouse, while the normal group (Normal) didn’t receive any administration. Normal, PBS, and BLM group received saline (1 ml/day) administered daily by intragastric administration, while BLM + WYHZTL-L group, BLM + WYHZTL-M group, BLM + WYHZTL-H group received low-dosage of WYHZTL formula (11.75 g/kg/d), medium-dosage of WYHZTL formula (23.50 g/kg/day), high-dosage of WYHZTL formula (47.00 g/kg/day) administered daily by intragastric administration of Bleomycin-induced SSc mouse models, respectively. BLM + XAV-939 group received XAV-939 (10 mg/kg/day) administered daily by intraperitoneal injection of Bleomycin-induced SSc mouse models. All the treatments lasted for 4 weeks, then the mice of each group were anaesthetized and then euthanized, and skin tissues were harvested, stored at − 80 °C, to analyze expression levels of target genes and proteins.

### Morphometric dermal measurements

The measurement of skin thickness, skinfold (pinch) thickness and breaking strength of skin were performed as previously described protocol [32]. Briefly, skin thickness was measured using a skin caliper on 12-mm punch biopsy samples obtained from the upper back, away from previous sites of drug injection. A skin caliper was used to measure skinfold (pinch) thickness at the same area of the upper back of the mouse. The measurement of breaking strength of skin was performed by a tensiometer, with a forceps clamped to one side of the 12-mm skin tissue sample and coupled to another forceps clamped to the furthermost extreme of the tissue sample. The value of maximal stress was recorded before tearing of the tissue sample [33]. All measurements were undertaken in a blinded manner.

### Histopathological analysis

Skin tissue samples were fixed with 4% paraformaldehyde, and embedded in paraffin, then cutted into 3 μm-thick sample sections for hematoxylin/eosin staining [34]. For hematoxylin/eosin stained tissue samples, a SCN400 Digital Slide Scanner (Leica Microsystems, Wetzlar, Germany) was used to examine and acquire images to identify histopathological changes. The diagnosis was performed by two independent examiners in a blinded manner.

### Western blotting analysis

The effects of the WYHZTL formula on Wnt/β-catenin signaling pathway was analyzed by Western blotting as previously described protocol [35]. Briefly, 1 day after WYHZTL formula and XAV-939 administration, the mice were killed to obtain skin tissues. Then the skin tissues were emulsified by ultrasound in RIPA lysis buffer (Beyotime Biotechnology, China). The protein concentrations were detected by BCA assay (Beyotime Biotechnology, China). 40 μg of protein was electrophoresed via sodium dodecyl sulphate polyacrylamide gel electrophoresis (SDS-PAGE), and transferred onto polyvinylidene fluoride (PVDF) membranes (Millipore, USA). Then the membranes were blocked using 5% skimmed milk powder at room temperature for 1 h. After blocking, membranes were incubated with specific antibodies against Wnt1 (1:500 dilution; abcam, UK), β-catenin, TCF4, DKK1, GSK3β, cyclin D1, survivin, CTGF, VEGF, Fibronectin, Collagen I, Collagen III (1:1000 dilution; Proteintech, USA), and GAPDH (1:5000 dilution; Proteintech, USA) overnight. After washing membranes with TBST buffer, membranes were incubated with corresponding horseradish peroxidase (HRP)-conjugated secondary antibody at a 1:5000 dilution for 1 h at 37 °C. Finally, the membranes were washed with TBST three times and developed using an BeyoECL Plus Kit by automated chemiluminescence system (Tanon, China) according to the manufacturer’s instructions. The optical density of immunoreactive bands was measured with a computer-assisted imaging analysis system (Tanon, China) and the relative protein expression levels were normalized to optical density of GAPDH.

### RT-qPCR analysis

Total RNA of each skin tissue samples was extracted using TRIzol reagent (Invitrogen, USA). All handling procedures perform under a laminar flow hood and RNAse-free conditions. The extracted RNA was dissolved in 30 μl RNase-free water and quantified by Nanodrop 2000 ultraviolet spectrophotometer (Thermo Fisher Scientific, Inc.), then stored at − 80 °C until used. RNA (2 µg) was reverse transcribed into complementary DNA (cDNA) using M-MLV Reverse Transcriptase (Thermo Fisher Scientific, Inc.) according to the manufacturer’s instructions. RT-qPCR analysis was performed using ABI Power SYBR Green PCR Master Mix Kit on an ABI Step One Plus instrument (Thermo Fisher Scientific, Inc.). The reaction condition follows denaturation for 2 min at 95 °C, 40 cycles including 95 °C for 15 s, 60 °C for 20 s and 72 °C for 30 s. Each experiment was performed 3 times independently. PCR products were mixed with 6× loading buffer (TaKaRa Biotechnology, Dalian, China) and then electrophoresed in 1.5% agarose gels containing ethidium bromide. The images were captured using a 4600SF Gel Image System (Tanon, China). The relative expression level of target gene was normalized to GAPDH and calculated with 2^−ΔΔCt^ method [36]. The primers for mRNA analysis for RT-qPCR were listed in the Table 1.

**Table 1:** Sequences of oligonucleotide primers and products size of RT-qPCR

mRNA	Primers (5′ → 3′) F: Forward R: Reverse	Product size (bp)
Wnt1	F: TGTTGACGGATTCCAAGAGT R: GAAGTAGACGAGGTCGTGAG	235
β-catenin	F: GGCAACCCTGAGGAAGAAGA R: AGCGTCAAACTGCGTGGAT	192
DKK1	F: ATCAATTCCAACGCGATCAA R: GGTCAGAGGGCATGCATATT	325
GSK3β	F: CCTGGTGCTGGACTATGT R: CAAGAGGTTCTGTGGTTTAA	214
TCF4	F: ACCACATGACTAGCAGGGATCT R: GGAGGAACTTTTCGGACTTTCT	298
VEGF	F: TTACTGCTGTACCTCCACC R: ACAGGACGGCTTGAAGATG	189
cyclin D1	F: GAGAAGTTGTGCATCTACAC R: GAAGGGCTTCAACTGTTCC	375
Survivin	F: GCTCTATGTAAGATCGCTTC R: CGTGAATCGCCATAATTATCC	389
CTGF	F: GTGTGTGACGAGCCCAAGGA R: AGTTGGGTCTGGGCCAAATGT	112
Fn1	F: ACCATACCTGCCGAATGTAG R: CTCCTCTCCAATGGCGTAAT	439
Collagen I	F: TGTTCGTGGTTCTCAGGGTAG R: TTGTCGTAGCAGGGTTCTTTC	245
Collagen III	F: TGCCCACAGCCTTCTACACCT R: CAGCCATTCCTCCCACTCCAG	254
GAPDH	F: TGGGTGTGAACCACGAGAA R: GGCATGGACTGTGGTCATGA	143

### Enzyme-linked immunosorbent assay (ELISA) analysis

One day after WYHZTL formula and XAV-939 administration, the animals were sacrificed. Collecting blood sample and standing to clot for 2 h at room temperature. Then centrifugation at 1000×*g* for 10 min, and obtain upper serum carefully to assay immediately. ELISA analysis was performed using enzyme-linked immunosorbent assay kit (Cusabio Technology, China) on a SpectraMax iD3 microplate reader instrument (Molecular Devices, USA). Reagents, and samples or standards were prepared, and experiments were performed according to the manufacturer’s instructions. Briefly, standard or sample were added to each well separately, and incubated at 37 °C for 2 h. Then standards or samples were removed without washing. Add Biotin-antibody to each well, and incubate at 37 °C for 1 h. Remove liquid of each well and wash. Add HRP-avidin to each well, and incubate at 37 °C for 1 h. Remove liquid of each well and wash. Add TMB Substrate to each well, and incubate at 37 °C for 20 min. Add Stop Solution to each well, and immediately detect the optical density of each well by a SpectraMax iD3 microplate reader instrument set to 450 nm. At last, the concentration of target protein was calculated according to standard curve. Each experiment was performed three times independently.

### Statistical analysis

Results were represented as mean ± SD. Data were analyzed by one-way analysis of variance, as appropriate, followed by Bonferroni post hoc test if F achieved *P* < 0.05 and there was no significant variance in homogeneity. All statistical analyses were performed with Graphpad Prism 6.0 Software. *P* < 0.05 was considered to indicate a statistically significant difference. All data and statistical analysis complied with the recommendations on experimental design and analysis in pathology.

## Results

### WYHZTL formula could ameliorate skin fibrosis in Bleomycin-induced SSc mouse model

**Fig. 1:**
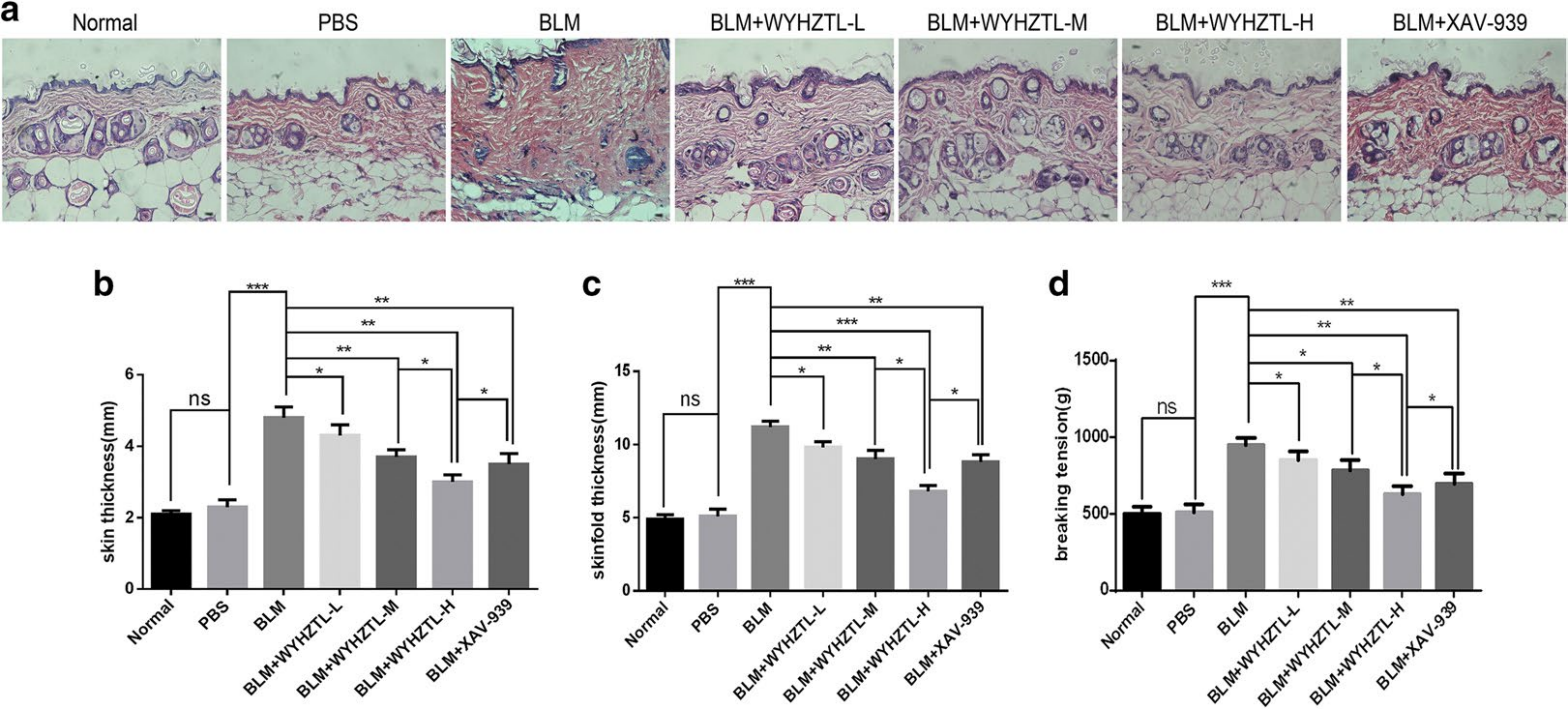
Bleomycin-induced SSc mouse model treated with WYHZTL formula and XAV-939 are protected against dermal fibrosis. Skin sections for histology were stained with hematoxylin and eosin (**a**). Representative photomicrographs are shown for each group. Differences in skin thickness (**b**), skinfold thickness (**c**), breaking tension (**d**) between Normal, PBS, BLM, BLM + WYHZTL-L (11.75 g/kg/day), BLM + WYHZTL-M (23.50 g/kg/day), BLM + WYHZTL-H (47.00 g/kg/day) or BLM + XAV-939 (10 mg/kg/day) groups after injections of Bleomycin (BLM) or phosphate buffered saline (PBS) for 3 weeks. The mean values ± SD (n = 8 per group) was shown for each bar. *(P < 0.05) or **(P < 0.01) or ***(P < 0.001) represents significance. Original magnification: ×20 (**a**). BLM: Bleomycin, WYHZTL: Wenyang Huazhuo Tongluo formula, WYHZTL-L: low-dosage of WYHZTL formula, WYHZTL-M: medium-dosage of WYHZTL formula, WYHZTL-H: high-dosage of WYHZTL formula

To confirm whether the Bleomycin-induced SSc mouse model was successfully constructed in this study, and detected the effect of WYHZTL formula and XAV-939 on skin fibrosis in mouse models, the skin of each mouse model group was dissected using paraffin sections, and the skin pathological changes of the mouse model were observed by hematoxylin and eosin stained (HE) staining. The results of HE staining showed that there was excessive accumulation of collagen and other extracellular matrix components in BLM group, which was attenuated with WYHZTL formula or XAV-939 treatment (Fig. 1a). The skin tissue of BLM group was significantly thicker than that of the PBS group. Compared with the BLM group, BLM + WYHZTL-L, BLM + WYHZTL-M, BLM + WYHZTL-H and BLM + XAV-939 groups displayed significantly reduced skin thickness (*P* < 0.05, *P* < 0.01, *P* < 0.01, *P* < 0.01, respectively) (Fig. 1b) and skinfold thickness (*P* < 0.05, *P* < 0.01, *P* < 0.001, *P* < 0.01, respectively) (Fig. 1c). These results were consistent with the higher dermal tensile strength, measured as breaking tension (*P* < 0.05, *P* < 0.05, *P* < 0.01, *P* < 0.01, respectively) (Fig. 1d). In addition, for skin thickness, skinfold thickness, and breaking tension, BLM + WYHZTL-H group showed more reduction than BLM + WYHZTL-L, BLM + WYHZTL-M, and BLM + XAV-939 groups (*P* < 0.05) (Fig. 1b–d). These results confirmed that WYHZTL formula or XAV-939 could significantly improve the thickening degree of the skin tissue, in addition, more importantly, the effect of WYHZTL formula was in a dosage-dependent manner, and WYHZTL-H had more advantage than XAV-939.

### Effect of WYHZTL formula on Wnt/β-catenin signal pathway in SSc mouse model

In order to determine whether the therapeutic effect of WYHZTL formula on SSc mouse model was related to Wnt/β-catenin signaling pathway, we collected the skin tissues of mice in each group, and detected the components of Wnt/β-catenin signal pathway using RT-qPCR and Western blotting.

**Fig. 2:**
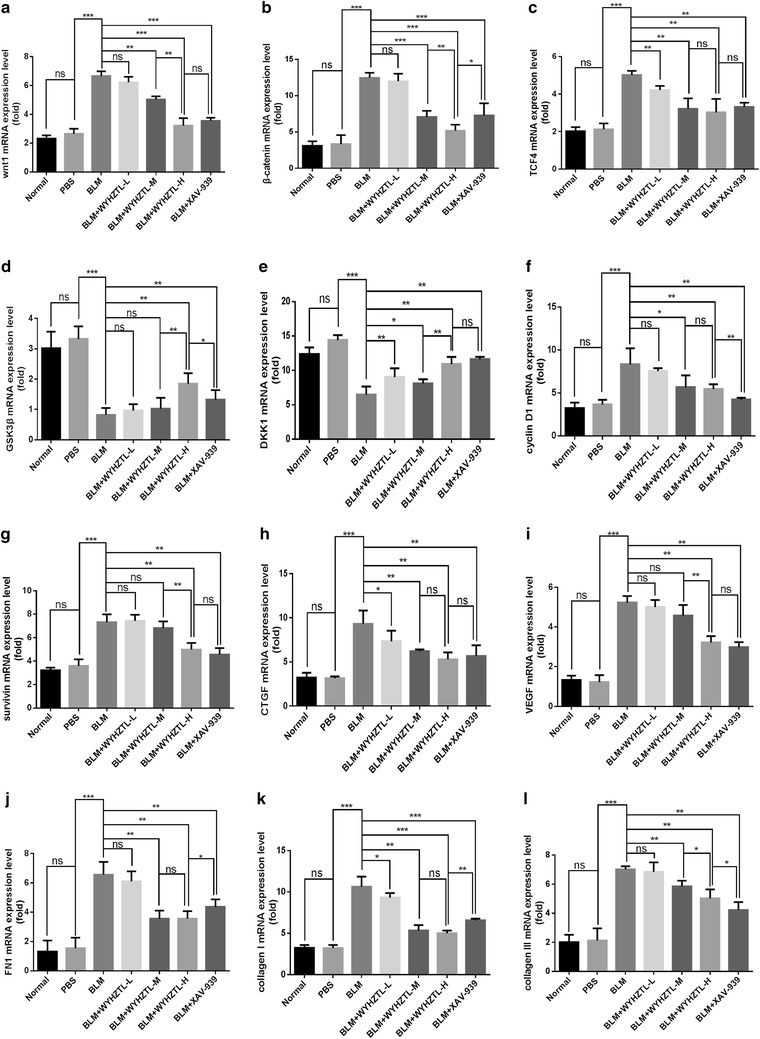
The ratios of the mRNA expression level of Wnt1, β-catenin, TCF4, GSK3β, DKK1, cyclin D1, survivin, CTGF, VEGF, FN1, Collagen I and Collagen III relative to that of GAPDH. The mRNA expression level ratios of Wnt1 (**a**), β-catenin (**b**), TCF4 (**c**), GSK3β (**d**), DKK1 (**e**), cyclin D1 (**f**), survivin (**g**), CTGF (**h**), VEGF (**i**), FN1 (**j**), Collagen I (**k**) and Collagen III (**l**) relative to GAPDH were calculated by RT-qPCR. The mean values ± SD (n = 8 per group) was shown for each bar. *(P < 0.05) or **(P < 0.01) or ***(P < 0.001) represents significance, ns represents no significance. BLM: Bleomycin, WYHZTL: Wenyang Huazhuo Tongluo formula, WYHZTL-L: low-dosage of WYHZTL formula, WYHZTL-M: medium-dosage of WYHZTL formula, WYHZTL-H: high-dosage of WYHZTL formula. RT-qPCR: Reverse transcription-quantitative polymerase chain reaction

The results of RT-qPCR showed that the mRNA expression level of GSK3β in BLM + WYHZTL-H, and BLM + XAV-939 group were significantly higher than that in BLM group (*P* < 0.01), while BLM + WYHZTL-L, BLM + WYHZTL-M groups had no significant changes (*P* > 0.05) (Fig. 2d). And the expression level of GSK3β in BLM + WYHZTL-H group was higher than BLM + WYHZTL-M, and BLM + XAV-939 group (*P* < 0.01, *P* < 0.05, respectively) (Fig. 2d). The expression level of DKK1 mRNA in BLM + WYHZTL-L, BLM + WYHZTL-M, BLM + WYHZTL-H, and BLM + XAV-939 group were significantly higher than that in BLM group (*P* < 0.01, *P* < 0.05, *P* < 0.01, *P* < 0.01, respectively) (Fig. 2e). And the expression level of DKK1 mRNA was higher than BLM + WYHZTL-L, BLM + WYHZTL-M group (*P* < 0.05, *P* < 0.01, respectively), rather than BLM + XAV-939 group (*P* > 0.05) (Fig. 2e). Compared to BLM group, the expression level of Wnt1 and β-catenin in BLM + WYHZTL-M, BLM + WYHZTL-H and BLM + XAV-939 group was significantly decreased (*P* < 0.01, *P* < 0.001, *P* < 0.001; all *P* < 0.001, respectively), while BLM + WYHZTL-L group had no significant changes (*P* > 0.05) (Fig. 2a, b). And the expression level of β-catenin in BLM + WYHZTL-H was lower than BLM + WYHZTL-M (*P* < 0.01) and BLM + XAV-939 (*P* < 0.05) group while the expression level of Wnt1 in BLM + WYHZTL-H were lower than BLM + WYHZTL-M (*P* < 0.01), other than BLM + XAV-939 group (*P* > 0.05) (Fig. 2a, b). Compared to BLM group, the expression level of TCF4 in BLM + WYHZTL-L, BLM + WYHZTL-M, BLM + WYHZTL-H and BLM + XAV-939 group was significantly decreased (*P* < 0.01) (Fig. 2c).

**Fig. 3:**
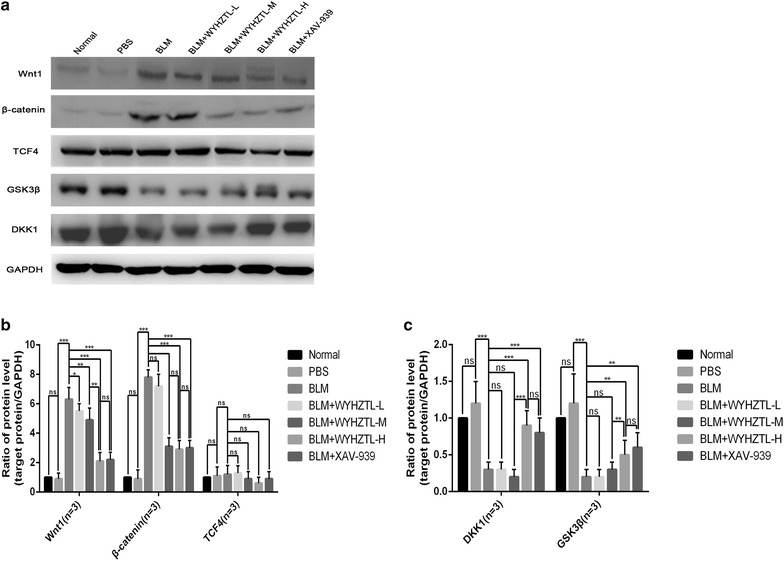
The ratios of the protein expression level of Wnt1, β-catenin, TCF4, GSK3β, DKK1 relative to GAPDH. **a** showed the Western blotting for expressions of Wnt1, β-catenin, TCF4, GSK3β, DKK1 and GAPDH. Representative results are shown. **b** and **c** showed the protein levels of Wnt1, β-catenin, TCF4, GSK3β, and DKK1 divided by the level of GAPDH. The mean values ± SD (n = 8 per group) was shown for each bar. *(P < 0.05) or **(P < 0.01) or ***(P < 0.001) represents significance, ns represents no significance. BLM: Bleomycin, WYHZTL: Wenyang Huazhuo Tongluo formula, WYHZTL-L: low-dosage of WYHZTL formula, WYHZTL-M: medium-dosage of WYHZTL formula, WYHZTL-H: high-dosage of WYHZTL formula

As shown in Fig. 3a, b, the result of Western blotting showed that compared with the BLM group, the expression level of Wnt1 protein in BLM + WYHZTL-L, BLM + WYHZTL-M, BLM + WYHZTL-H, and BLM + XAV-939 group were significantly decreased, while BLM + WYHZTL-H was even lower than BLM + WYHZTL-M group, other than BLM + XAV-939 group. Compared with the BLM group, the expression level of β-catenin protein in BLM + WYHZTL-M, BLM + WYHZTL-H, and BLM + XAV-939 group were significantly decreased, while BLM + WYHZTL-L had no significant changes (*P* > 0.05), and among BLM + WYHZTL-M, BLM + WYHZTL-H, and BLM + XAV-939 group, there were no significant difference (*P* > 0.05). However, in this study, we didn’t detect any significant difference among Normal group, PBS group and BLM group. And WYHZTL formula or XAV-939 also had no effect on the expression level of TCF4 protein.

As shown in Fig. 3a, c, the expression level of DKK1 and GSK3β protein in BLM + WYHZTL-H and BLM + XAV-939 group were significantly higher than that in BLM group (*P* < 0.001, *P* < 0.01, respectively), while BLM + WYHZTL-L and BLM + WYHZTL-M group didn’t have significant difference with BLM group (*P* > 0.05). And the expression level of DKK1 and GSK3β protein in BLM + WYHZTL-H were all higher than BLM + WYHZTL-M group (*P* < 0.001, *P* < 0.01, respectively), but not BLM + XAV-939 group (*P* > 0.05).

### The effect of WYHZTL formula on target genes of Wnt/β-catenin signaling pathway and fibrosis marker

In order to further study on regulating SSc fibrosis by WYHZTL formula via Wnt/β-catenin signaling pathway, we investigated the expression level of target genes of Wnt/β-catenin signaling pathway and fibrosis markers in the skin tissues of each mouse model by RT-qPCR and Western blotting.

The results of RT-qPCR showed that compared with the BLM group, the mRNA expression levels of cyclin D1, FN1 and collagen III in BLM + WYHZTL-M, BLM + WYHZTL-H and BLM + XAV-939 group were downregulated (*P* < 0.05, *P* < 0.01, *P* < 0.01; all *P* < 0.01; all *P* < 0.01, respectively), while BLM + WYHZTL-L group didn’t significantly change (*P* > 0.05) (Fig. 2f, j and l). Compared with the BLM group, the mRNA expression levels of survivin and VEGF in BLM + WYHZTL-H and BLM + XAV-939 group were downregulated (all *P* < 0.01), while BLM + WYHZTL-L and BLM + WYHZTL-M group didn’t significantly change (*P* > 0.05) (Fig. 2i). The mRNA expression levels of CTGF and collagen I in BLM + WYHZTL-L, BLM + WYHZTL-M, BLM + WYHZTL-H and BLM + XAV-939 group were all lower than BLM group (*P* < 0.05, *P* < 0.01, *P* < 0.01, *P* < 0.01; *P* < 0.05, *P* < 0.01, *P* < 0.001, *P* < 0.001, respectively) (Fig. 2h, k).

In addition, the mRNA expression levels of survivin, VEGF and collagen III in BLM + WYHZTL-H group were even lower than BLM + WYHZTL-M group (*P* < 0.01, *P* < 0.01, *P* < 0.05, respectively), while the mRNA expression levels of FN1, collagen I in BLM + WYHZTL-H group were even lower than BLM + XAV-939 group (*P* < 0.05, *P* < 0.01, respectively) (Fig. 2g, i–l). Besides that, the mRNA expression levels of cyclin D1 and collagen III in BLM + XAV-939 group were lower than BLM + WYHZTL-H group (*P* < 0.01, *P* < 0.05, respectively) (Fig. 2f, l).

**Fig. 4:**
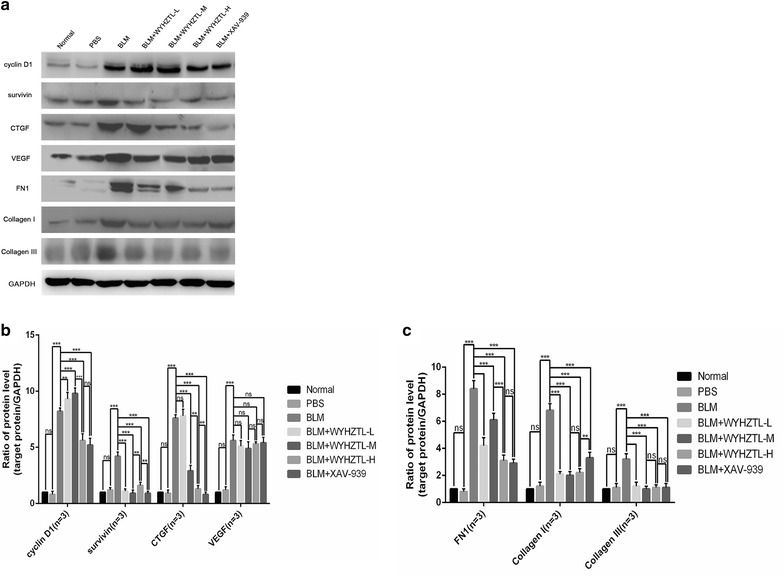
The ratios of the protein expression level of cyclin D1, survivin, CTGF, VEGF, FN1, Collagen I and Collagen III relative to GAPDH. **a** showed the Western blotting for expressions of cyclin D1, survivin, CTGF, VEGF, FN1, Collagen I, Collagen III and GAPDH. Representative results are shown. **b** and **c** showed the protein levels of cyclin D1, survivin, CTGF, VEGF, FN1, Collagen I, Collagen III divided by the level of GAPDH. The mean values ± SD (n = 8 per group) was shown for each bar. ***(P < 0.001) represents significance, ns represents no significance. BLM: Bleomycin, WYHZTL: Wenyang Huazhuo Tongluo formula, WYHZTL-L: low-dosage of WYHZTL formula, WYHZTL-M: medium-dosage of WYHZTL formula, WYHZTL-H: high-dosage of WYHZTL formula

As shown in Fig. 4a, b and c, the results of Western blotting showed that compared with the BLM group, the protein expression levels of cyclin D1, survivin, FN1 and collagen I/III in BLM + WYHZTL-L, BLM + WYHZTL-M, BLM + WYHZTL-H and BLM + XAV-939 group were all downregulated (*P* < 0.01, *P* < 0.001, *P* < 0.001, *P* < 0.001; all *P* < 0.001; all *P* < 0.001; all *P* < 0.001, respectively), while CTGF in BLM + WYHZTL-M, BLM + WYHZTL-H and BLM + XAV-939 group was decreased (all *P* < 0.001). In addition, the protein expression levels of cyclin D1, CTGF, FN1 in BLM + WYHZTL-H group were even lower than BLM + WYHZTL-M group (all *P* < 0.001), while the protein expression levels of collagen I in BLM + WYHZTL-H group was lower than BLM + XAV-939 group (*P* < 0.01). Besides that, the protein expression levels of CTGF in BLM + XAV-939 group was even lower than BLM + WYHZTL-H group (*P* < 0.01).

Furthermore, compared to Normal and PBS group, we found that VEGF was upregulated in BLM group (*P* < 0.001), but there was no significant difference among BLM, BLM + WYHZTL-L, BLM + WYHZTL-M, BLM + WYHZTL-H and BLM + XAV-939 group (*P* > 0.05).

### Effect of WYHZTL formula on the protein level of Wnt-1, DKK1 and CTGF in serum of SSc mouse model

In order to further identify the regulation of Wnt/β-catenin signaling pathway by WYHZTL formula, we collected the serum of each group of mouse model and detected the protein level of Wnt1, DKK1 and CTGF in serum by ELISA.

**Fig. 5:**
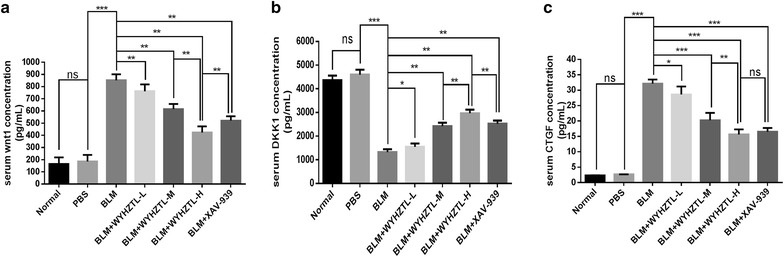
The protein expression level of Wnt1, DKK1 and CTGF in serum. The protein concentrations of Wnt1 (**a**), DKK1 (**b**) and CTGF (**c**) were detected by ELISA. The mean values ± SD (n = 8 per group) was shown for each bar. *(P < 0.05) or **(P < 0.01) or ***(P < 0.001) represents significance, ns represents no significance. BLM: Bleomycin, WYHZTL: Wenyang Huazhuo Tongluo formula, WYHZTL-L: low-dosage of WYHZTL formula, WYHZTL-M: medium-dosage of WYHZTL formula, WYHZTL-H: high-dosage of WYHZTL formula. *ELISA* enzyme-linked immunosorbent assay

As shown in Fig. 5a, b and c, compared with BLM group, the protein level of Wnt1 and CTGF in BLM + WYHZTL-L, BLM + WYHZTL-M, BLM + WYHZTL-H and BLM + XAV-939 group were significantly downregulated (all *P* < 0.01; *P* < 0.05, *P* < 0.001, *P* < 0.001, *P* < 0.001, respectively), while DKK1 was upregulated (*P* < 0.05, *P* < 0.01, *P* < 0.01, *P* < 0.01, respectively), in which WYHZTL-H became more effective on promoting DKK1 (*P* < 0.001), while inhibiting Wnt1 and CTGF (all *P* < 0.001) than WYHZTL-M. Furthermore, compared with BLM + XAV-939 group, WYHZTL-H was more effective in downregulating Wnt1, and upregulating DKK1 (all *P* < 0.001).

## Discussion

Wnt/β-catenin signaling pathway could induce fibrosis. Wnt1 and human skin fibroblasts were co-incubated to significantly stimulate the expression of α-SMA and type II collagen, which meant that Wnt1 could induce the transformation of resting fibroblasts to myofibroblasts, and stimulate collagen release [18, 37]. Previous works exhibited that Wnt signal overactivates in SSc patients which played an important role in myofibroblast differentiation and fibroblast proliferation, and also inducing adipose precursor cells transform to fibroblasts by DNA chip analysis and immunohistochemistry technology [38, 39]. At the same time, overexpressing Wnt-10b in normal fibroblasts could upregulate the expression level of fibrosis-related genes [19, 40]. In vivo experiments had also shown that Wnt-10b transgenic mice appear skin fibrosis, progressive subcutaneous fat loss, collagen accumulation, fibroblast activation, myofibroblast aggregation and other phenomena, while Wnt/β-catenin signaling pathway was overactivated in skin fibroblasts [19, 41], which further confirmed that activation of Wnt/β-catenin signaling pathway could induce fibrosis. In the present study, we also found that Wnt/β-catenin signaling pathway was overactivated in the BLM-induced SSc mouse model, suggesting that Wnt/β-catenin signaling pathway indeed played an important role in the pathogenesis of SSc. More importantly, WYHZTL formula could significantly improve skin fibrosis in SSc mouse model. Further study found that WYHZTL formula could inhibit the Wnt/β-catenin signaling pathway. These results suggested that WYHZTL formula may exert anti-SSc fibrosis effect by inhibiting Wnt/β-catenin signaling pathway.

Fibrosis, atrophy and vascular occlusive vasculitis of connective tissue were basic pathological changes of SSc [6]. Fibrosis may involve multiple visceral systems, such as the gastrointestinal tract, lung, kidney, heart, blood vessels, skeletal muscle system, causing dysfunction of corresponding organs [42]. Among them, the skin tissue fibrosis and dermal thickening were the most important and common pathological features of SSc. Yamamoto and colleagues [43] used the Bleomycin (BLM) treatment to establish a new mouse model of SSc: subcutaneous injection of BLM can induce dermal and pulmonary fibrosis, autoantibody production and skin inflammatory infiltration, which was one of the best animal models for studying on systemic sclerosis. In this study, we used Bleomycin-induced mice on a C57BL/6 background to construct systemic sclerosis model. We identified Bleomycin-induced mouse skin tissue by HE staining, showing that fibrosis, dermal thickening, inflammatory cell infiltration and other pathological changes, suggesting that Bleomycin-induced systemic sclerosis mouse model was successfully constructed. Then we treated the SSc mouse model with WYHZTL formula and XAV-939, respectively. The result of HE staining showed that compared with the model group, the thickening degree of skin of the SSc mouse model was significantly thinner in WYHZTL formula and XAV-939 group, moreover, we found that the effect of WYHZTL formula was more significant than XAV-939.

The overexpression of target genes of Wnt/β-catenin signaling pathway were closely related to SSc fibrosis. The target genes include c-myc, cyclin D1, survivin, vascular endothelial growth factor (VEGF), transforming growth factor-β (TGF-β), connective tissue growth factor (CTGF), endothelin-1 (ET-1), and extracellular matrix components, such as fibronectin (FN) [44]. Therefore, Wnt/β-catenin signaling pathway may be acted as an important target for the treatment of SSc fibrosis. It may be one of effective ways to treat SSc fibrosis by regulating Wnt/β-catenin signaling pathway and its target genes [24]. In our previous study, we had found WYHZTL formula could inhibit the expression of cyclin D1 and survivin in skin fibroblasts of SSc patients [29]. The results of this study showed that WYHZTL formula could upregulate the mRNA expression of GSK3β and DKK1, while downregulated Wnt1, β-catenin, TCF4, cyclin D1, survivin, VEGF, CTGF, FN1, Type I/III collagen. More importantly, after treatment of WYHZTL formula, the protein expression level of DKK1 was increased, while the protein expression levels of Wnt1, β-catenin, cyclin D1, survivin, CTGF, FN1, collagen I/III were decreased. In addition, it was interesting that we also found that the regulation of Wnt/β-catenin signaling pathway and fibrotic-related protein by WYHZTL formula was in a dosage-dependent manner, which was to say, high-dosage of WYHZTL formula seemed more effective than low-dosage or medium-dosage. This discovery prompted us to consider the impact of dosage when we applied WYHZTL formula to the SSc treatment. These results suggested that WYHZTL formula could inhibit fibrosis in SSc by regulating Wnt/β-catenin signaling pathway, which was consistent with our previous findings in SSc fibroblasts [29].

In addition, considering that Wnt1, DKK1 and CTGF are secreted proteins, we detected the protein expression levels of Wnt1, DKK1 and CTGF in serum of mice by ELISA. We found that WYHZTL formula and XAV-939 could significantly reduce the protein level of Wnt1 and CTGF, while increase the protein level of DKK1 in serum, which was consistent with the results in the study of skin tissue, suggesting that WYHZTL formula could indeed inhibit the development of fibrosis by regulating Wnt/β-catenin signal pathway.

XAV-939 was widely used as an inhibitor of Wnt/β-catenin signaling pathway [45]. Our previous studies [29] also demonstrated that XAV-939 inhibit expression of cyclin D1 and survivin, target genes of Wnt/β-catenin signaling pathway, in SSc fibroblasts. In this study, we firstly applied XAV-939 to the SSc mouse model. The results showed that XAV-939 could significantly inhibit Wnt/β-catenin signaling pathway and fibrotic-related protein in vivo. More importantly, our results suggested that WYHZTL formula was more effective than XAV-939, that was to say, WYHZTL formula could inhibit Wnt/β-catenin signal pathway as XAV-939 worked in treatment of SSc mouse model, but WYHZTL formula may have another mechanism of anti-fibrosis except Wnt/β-catenin signal pathway. As was known to all, in addition to the Wnt/β-catenin signaling pathway, there was another signaling pathway that also played an important role in the pathogenesis of SSc, such as the TGF-β/smad signaling pathway [46]. In our previous study, we had found that WYHZTL formula could inhibit TGF-β/smad signaling pathway, thereby inhibiting collagen expression in SSc skin fibroblasts and exerting anti-fibrosis effect [30]. So this may explain that in our study, although the effect of low-dosage of WYHZTL formula on Wnt/β-catenin signaling pathway was not obvious, it may exert anti-SSc fibrosis through other mechanisms, such as TGF-β/smad signaling pathway. In addition, autoimmune abnormality was also an important marker of SSc and play a key role in the development of SSc [47]. The level of IL-17A expression was positively correlated with skin and lung inflammation scores and skin fibrosis in BLM-induced mice model. IL-17A could significantly enhance the proliferation of lung fibroblasts in vitro [48]. And Th17 cells in peripheral blood, skin and lung tissue of SSc mouse model were significantly increased, and were closely related to the inflammation and fibrosis of skin and lung tissue [49]. In our previous study, we had found WYHZTL formula could achieve its therapeutic effect on SSc patients by regulating Th17/Treg imbalance and inhibit the expression of IL-17 [31]. All of these results showed that WYHZTL formula worked by many mechanisms, except for regulating Wnt/β-catenin signaling pathway, suggesting that the effect of WYHZTL formula in SSc treatment should be multi-mechanism and multi-level.

## Conclusions

**Fig. 6:**
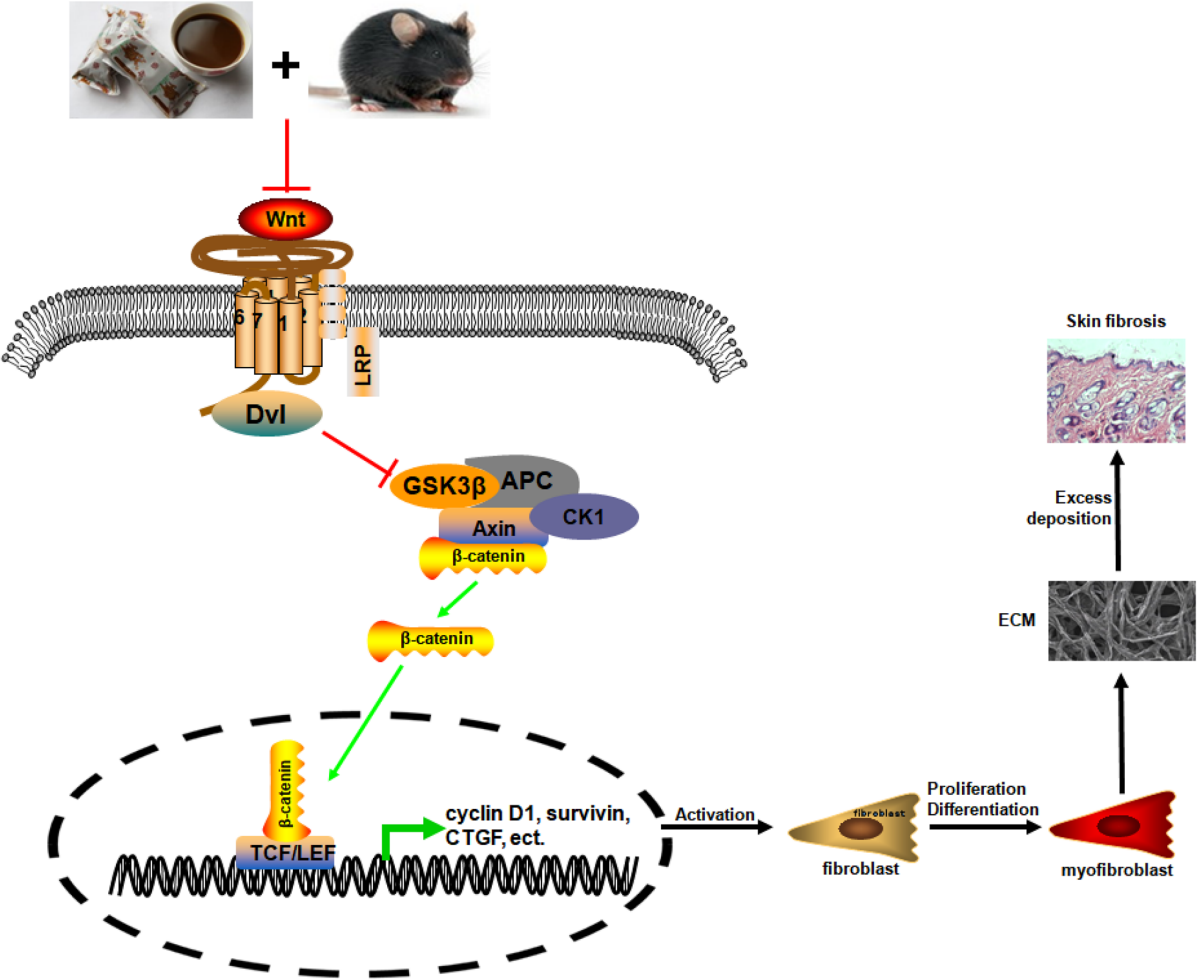
A schematic diagram illustrating the mechanism of WYHZTL formula in regulating Wnt/β-catenin signaling pathway. When Wnt protein binds to the transmembrane frizzled receptor (Frizzled family member, Frz) and co-receptor LRP on the cell membrane, cytoplasmic disheveled (Dsh) inhibits activity of the Glycogen synthase kinase 3β (GSK3β) which causes the depolymerization of β-catenin destruction complex-Axin/APC/casein kinase Iα (CKIα)/GSK3β. The degradation of β-catenin is inhibited, then enriched in the cytoplasm and entered into the nucleus, which interacts with transcription factor T cell factor/lymphoid enhancer (TCF/LEF) to form a transcription complex that initiates downstream target genes expression. The target genes include cyclin D1, survivin, connective tissue growth factor (CTGF), which results in activation and transformation of fibroblast to myofibroblast. At last, the excess deposition of extracellular matrix (ECM) leads to the occurrence of skin fibrosis. In our present study, we reveal a mechanism of WYHZTL formula in inhibiting Wnt/β-catenin signaling pathway which can ameliorate skin fibrosis in Bleomycin-induced SSc mouse model

In conclusion, our results validated the function of anti-fibrosis in SSc treatment for WYHZTL formula, and also proposed a new molecular mechanism of anti-fibrosis by inhibiting Wnt/β-catenin signaling pathway in Bleomycin-induced SSc mouse model (Fig. 6). At present, WYHZTL formula is widely used in the treatment of SSc, and our results provides a scientific explanation for its treatment mechanism.

## Additional file

**Additional file 1.** Minimum Standards of Reporting Checklist.

## Abbreviations

SSc: systemic sclerosis
WYHZTL: Wenyang Huazhuo Tongluo formula
DKK1: Dickkopf 1
CTGF: connective tissue growth factor
GSK3β: glycogen synthase kinase 3β
TCF4: T cell factor 4
VEGF: vascular endothelial growth factor
FN: fibronectin
APC: adenomatous polyposis coli
CK1: casein kinase 1
ET-1: endothelin-1
MMP-9: matrix metalloproteinase-9
GAPDH: glyceraldehyde-3-phosphate dehydrogenase
TIMP-1: tissue inhibitor of metalloproteinases-1
PIIINP: procollagen III N-terminal peptide
ICTP: I-collagen carboxyl terminal telopeptide
ECM: excessive accumulation of extracellular matrix
FVC: forced vital capacity
DLco: carbon monoxide diffusing capacity
ELISA: enzyme-linked immunosorbent assay
Ct: cycle threshold
RT-qPCR: reverse transcription-quantitative polymerase chain reaction
